# Supplementary material for: Radiative anti-parity-time plasmonics
Source: Nat Commun. 2022 Dec 12;13:7678. doi: 10.1038/s41467-022-35447-3 (PMC9744817; doi:10.1038/s41467-022-35447-3)
Supplement: Supplementary file 1 — Supplementary Information [file 41467_2022_35447_MOESM1_ESM.pdf]

Supplementary Information for

Radiative Anti-Parity-Time Plasmonics

Yumeng Yang[1,2,3,4]†, Xinrong Xie[1,2,3,4]†, Yuanzhen Li[1,2,3,4], Zijian Zhang[1,2,3,4], Yiwei Peng[1,2,3,4], Chi Wang[1,2,3,4], Erping Li[1,2,3,4], Ying Li[1,2,3,4], Hongsheng Chen[1,2,3,4*], Fei Gao[1,2,3,4*]

[1]Interdisciplinary Center for Quantum Information, State Key Laboratory of Extreme Photonics and Instrumentation, ZJU-Hangzhou Global Scientific and Technological Innovation Center, Zhejiang University, Hangzhou 310027, China.

[2]International Joint Innovation Center, The Electromagnetics Academy at Zhejiang University, Zhejiang University, Haining 314400, China.

[3]Key Lab. of Advanced Micro/Nano Electronic Devices & Smart Systems of Zhejiang, Jinhua Institute of Zhejiang University, Zhejiang University, Jinhua 321099, China.

[4]Shaoxing Institute of Zhejiang University, Zhejiang University, Shaoxing 312000, China.

†Authors contributed equally to this work

*Corresponding author. Email: hansomchen@zju.edu.cn (H. Chen), gaofeizju@zju.edu.cn (F. Gao)

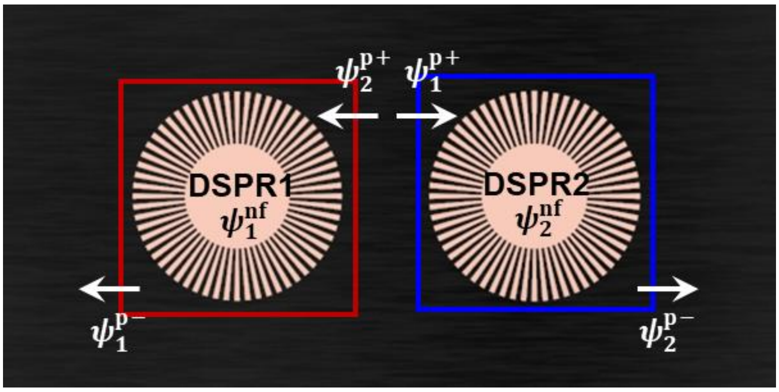

Supplementary Figure 1. The indirectly coupled designer-plasmonic APT system. The incoming propagating waves and outgoing waves are $\psi_{1(2)}^{p+}$ and $\psi_{1(2)}^{p-}$, respectively.

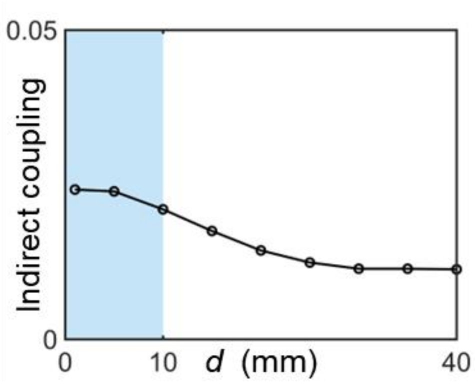

Supplementary Figure2. The simulated indirect coupling strength χ as the coupling distance *d* between the two DSPRs evolves.

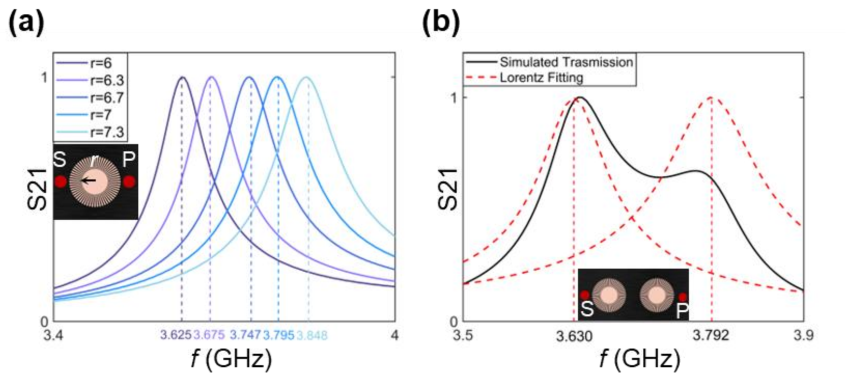

Supplementary Figure 3. Parameter extraction. (**a**) The simulated transmission spectra of single DSPR with different inner radii *r*. The inset shows the setup in simulations. The ‘S’ and ‘P’ denote the locations of the near-field source and probe respectively. (**b**) The simulated transmission spectrum (black line) of two indirectly coupled DSPRs with r_R = 6 mm, r_L = 7 mm and d = 10 mm. The red lines denote the Lorentz fitting results. The inset shows the simulation setup.

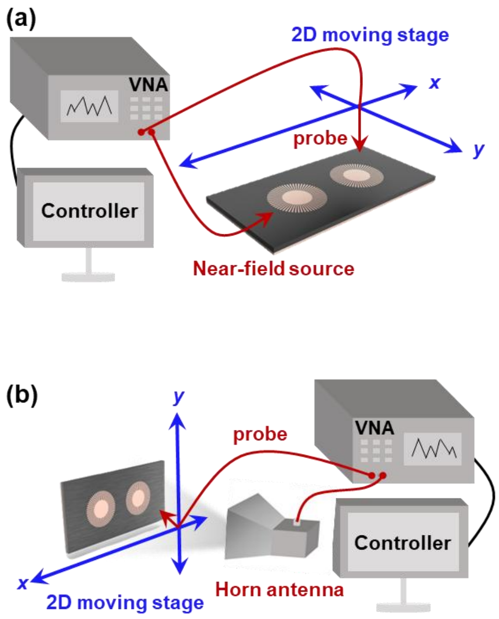

Supplementary Figure 4. Schematics of near-field and far-field experimental setup. (**a**) Near-field experimental setup. (**b**) Far-field experimental setup.

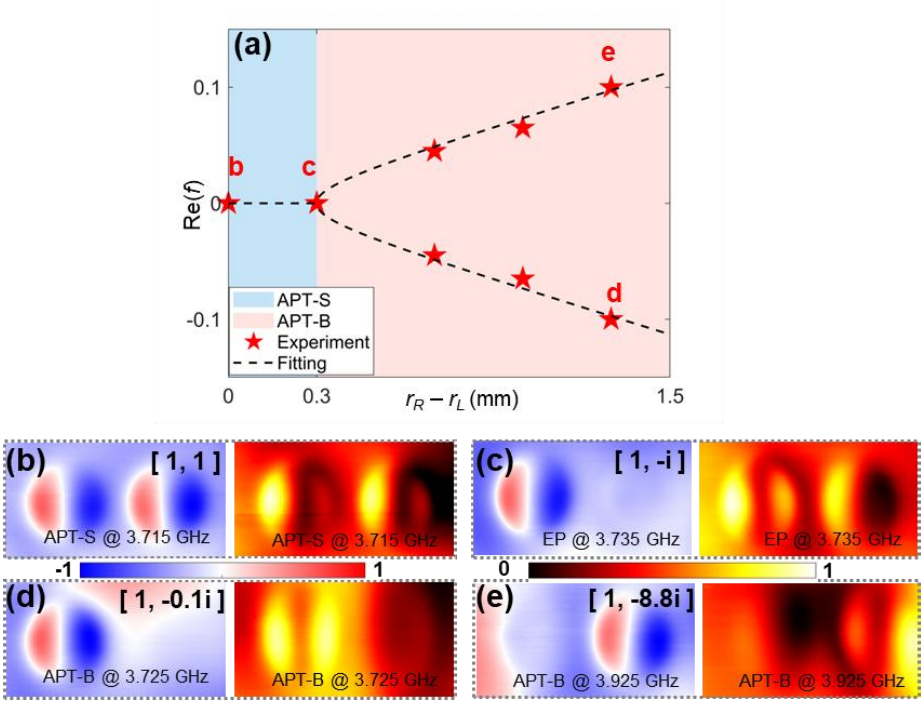

Supplementary Figure 5. The detuning-induced APT phase transition under space-wave illuminations. (**a**) The evolution of eigenfrequencies as $r_L - r_R$ changes. The red stars mark the detected resonance frequencies. (**b-e**) The field patterns E_z by far-field excitation and near-field imaging, correspond to APT-S ($|\delta/\kappa| = 0$), EP ($|\delta/\kappa| = 1$), and APT-B phases ($|\delta/\kappa| = 4.46$), respectively. The distributions of E_z and $|E_z|$ are shown with rainbow and hot colors, respectively.

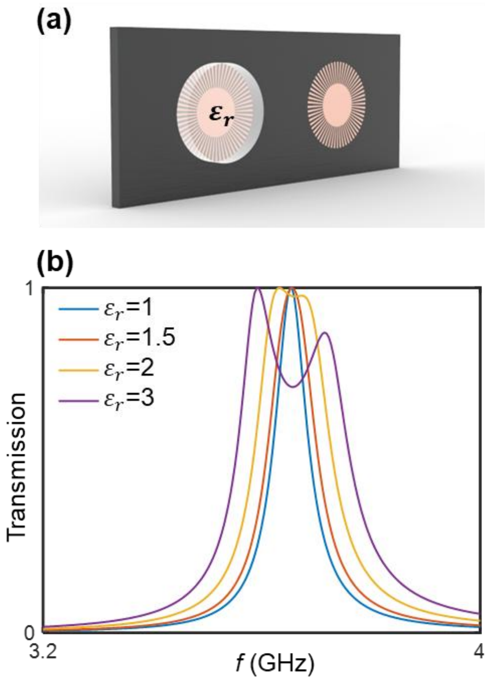

Supplementary Figure 6. Detection of environmental variations. (**a**) The plasmonic APT system for environmental variation detection. The inner radius of the left and right DSPRs is 6 mm. The left DSPR is covered with the media of the relative permittivity ε_r. (**b**) The evolution of the simulated transmission spectra as the media permittivity ε_r changes.

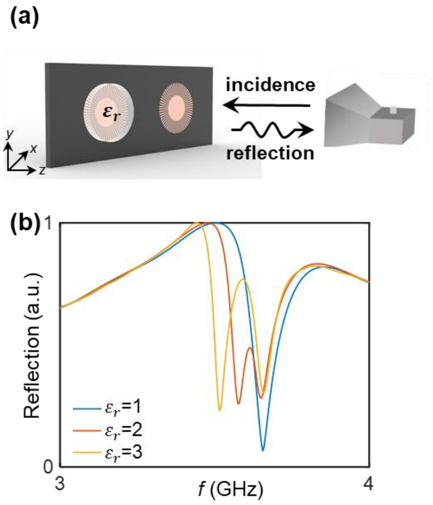

Supplementary Figure 7. Remote sensing of environmental variations. (**a**) The simulation setup for remote sensing of environmental variations. (**b**) The evolution of the simulated reflection spectra as the media permittivity ε_r changes.

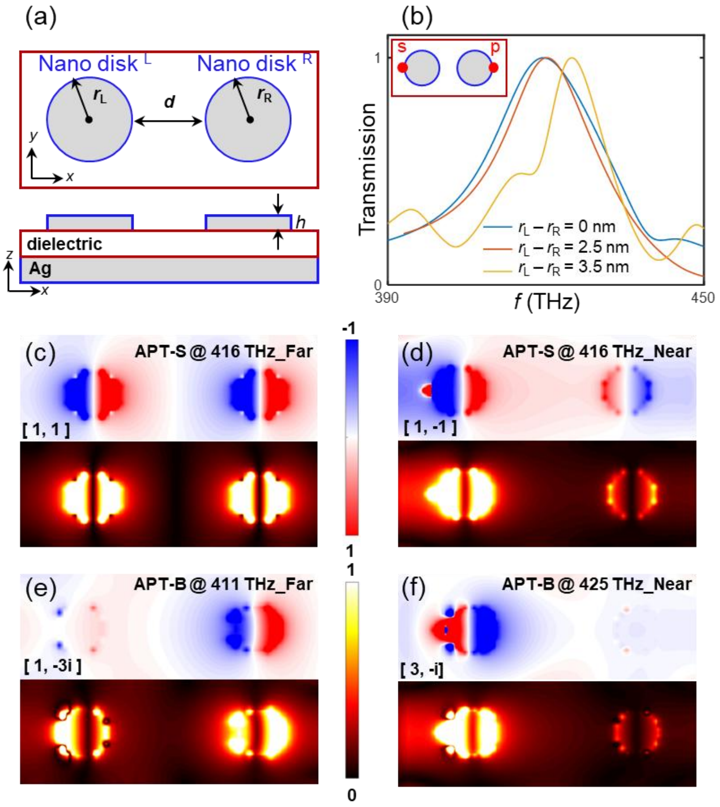

Supplementary Figure 8. The extension of APT design to optical frequencies. (**a**) The top and the front view of the plasmonic APT system. It consists of two silver (Ag) nanodisks with a distance d = 170 nm. The radii of the two nanodisks are r_R (right) and r_L (left), and the thickness h is 20 nm. The background structure is composed of two layers. The top layer is a dielectric layer (ε_r = 1.5) of 50 nm thickness. The bottom layer is a silver substrate of 20 nm thickness. (**b**) The simulated evolution of the transmission spectra as $r_L - r_R$ varies. The inset shows the simulation setup. (**c-d**) The field patterns E_z correspond to the APT-S phase ($r_L - r_R = 0$). (**e-f**) The field patterns E_z correspond to the APT-B phase ($r_L - r_R$ = 3.5 nm). The distributions of E_z and $|E_z|$ are shown with rainbow and hot colors.

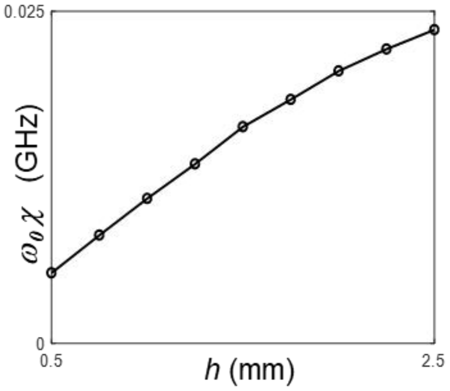

Supplementary Figure 9. The evolution of the imaginary coupling strength χ as the thickness h of the substrate changes.

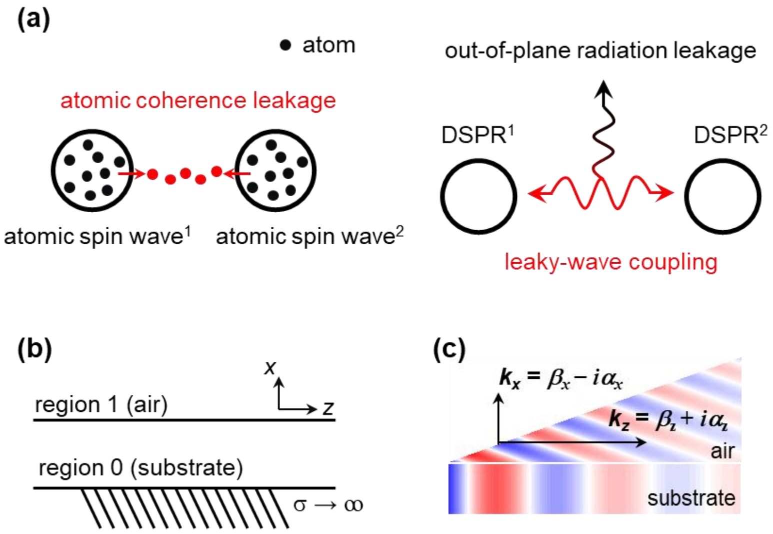

Supplementary Figure 10. Different physical properties of the atomic coherence leakage and the leaky wave. (**a**) Schematics of the atomic coherence leakage[1] and leaky wave. (**b**) The typical structure of the leaky wave. (**c**) The field pattern of the leaky wave.

	Atomic coherence leakage[1]	Leaky wave
Type	Atoms (quantum wave)	EM wave (classical wave)
Non-Hermitian mechanism	Randomness & irreversibility	Out-of-plane leakage
Property	N.A.	1. Polarization response to space wave<br>2. Parity modulation

Supplementary Table 1. Different physical properties of the atomic coherence leakage and the leaky wave.

Supplementary Note I

Theoretical model

Regarding the designer-plasmonic APT system consisting of two indirectly coupled DSPRs as shown in Fig. S1, we utilize coupled-mode equations to describe the system as follows

$$\begin{cases} i\frac{\mathrm{d}}{\mathrm{dt}}\psi = (\Omega + i\gamma)\psi + \ i\tau l \\ \qquad l = \ D\psi \\ \qquad s^{-} = \ C\psi \end{cases} \tag{1.1}$$

The basis is $\psi = [\psi_1^{\mathrm{nf}}, \ \psi_2^{\mathrm{nf}}]^{\mathrm{T}}$, where elements are localized resonant modes of DSPRs. The diagonal and off-diagonal terms of $\Omega = [\omega_1, \ \kappa_{12}; \ \kappa_{21}, \ \omega_2]$ denote the resonance frequencies and coefficients of direct couplings. $\gamma = [\gamma_1, 0; 0, \gamma_2]$ means the decay rate. The matrix $\tau = [0, \ \tau_{12}; \ \tau_{21}, 0]$ represents the indirect coupling coefficient through continuum channels $l = [\psi_1^{\mathrm{p}+}, \ \psi_2^{\mathrm{p}+}]^{\mathrm{T}}$. The continuum channels are propagating waves generated by localized resonant modes with the efficiency $D = [D_0, 0; 0, D_0]$. In addition, the localized modes also leak energy into the environment through the outgoing waves $s^{-} = [\psi_1^{\mathrm{p}-}, \ \psi_2^{\mathrm{p}-}]^{\mathrm{T}}$ with leaky rate $C = [c_1, c_2; c_2, c_1]$. By simple manipulation, Eq.1.1 can be written in a compact form

$$\begin{cases} i\frac{\mathrm{d}}{\mathrm{d}t}\psi = (\Omega + i\Gamma)\psi \\ \qquad s^{-} = \ C\psi \end{cases} \tag{1.2}$$

where $\Gamma = [\gamma_1, \chi_{12}; \chi_{21}, \gamma_2]$, and $\chi_{12} = \tau_{12}D_0$ ($\chi_{21} = \tau_{21}D_0$). Parameters in the equation are not independent, and their relations rely upon energy conservation and reciprocity[2].

Since the whole system, including both the resonators and the environment, is energy conserved, the decay of the resonance modes is entirely due to the outgoing waves. Therefore, the following equation is obtained based on energy conservation

$$\frac{\mathrm{d}\psi^{+}\psi}{\mathrm{d}t} = -< s^{-}|s^{-} > \tag{1.3}$$

We consider the scenario where the resonant modes have finite amplitudes at $t = 0$. At $t > 0$, the resonant modes will decay into the environment through the outgoing waves. In such a scenario, substituting Eq.1.2 with Eq.1.3, we have

$$C^{+}C = \ 2\Gamma \tag{1.4}$$

Therefore, we obtain $\Gamma_{+} = \Gamma_{-}$, thus $\chi_{12} = \chi_{21}$.

Supplementary Note II

Indirect coupling strength

The indirect coupling through leaky waves is elucidated as $i\chi \propto h\cdot\int(\psi_{1(2)}^{\mathrm{nf}}\cdot\psi_{2(1)}^{\mathrm{p}})/(|C_{\mathrm{NF}}|\cdot|C_{\mathrm{FF}}|)\mathrm{d}x\mathrm{d}y$. The field distributions of ψ^{nf} is approximated as $\cos(\pi y/2R)\sin(\pi x/2R)$ (odd) or $\sin(\pi y/2R)\cos(\pi x/2R)$ (even). Due to the parity modulation by ψ^{nf}, ψ^{p} is approximated as $\cos(\pi y/2R)e^{ikx}$ (odd) or $\sin(\pi y/2R)e^{ikx+i\pi/2}$ (even), respectively. Therefore, the non-vanished indirect coupling gives pure imaginary values as:

$$i\chi_{\mathrm{o}} \propto \left(\frac{\frac{\pi y}{R}+\sin\left(\frac{\pi y}{R}\right)}{\frac{\pi}{2R}}\right) * \left(\frac{\pi/2R\,(\sin(kx)\cos\left(\frac{\pi x}{2R}\right))-k\,(\sin\left(\frac{\pi x}{2R}\right)\cos(kx))}{(k)^2-\left(\frac{\pi}{2R}\right)^2}\right) \text{ (odd)}$$

$$i\chi_{\mathrm{e}} \propto \left(\frac{\frac{\pi y}{R}-\sin\left(\frac{\pi y}{R}\right)}{\frac{\pi}{2R}}\right) * \left(\frac{k\,(\sin(kx)\cos\left(\frac{\pi x}{2R}\right))-\pi/2R\,(\sin\left(\frac{\pi x}{2R}\right)\cos(kx))}{(k)^2-\left(\frac{\pi}{2R}\right)^2}\right) \text{ (even)}$$

which is independent of the distance d between two resonators.

We further extract the indirect coupling strength from simulation results (in Fig. S2) as the coupling distance d changes. When the distance increases from 1 mm to 10 mm, the variation range of the indirect coupling strength $\omega_0\chi$ is only 0.003 GHz (from 0.024 GHz to 0.021GHz). Such a small variation is negligible, so the indirect coupling strength can be approximately considered stable in the proper region of 1-10 mm (the blue region in Fig. S2).

As the coupling distance d further increases, the simulated indirect coupling strength decreases slightly. Such a deviation from theoretical derivation is attributed to the attenuation of the in-plane propagating component of leaky waves in the presence of the out-of-plane radiation leakage.

Supplementary Note III

Eigen solutions of the APT Hamiltonian

Solving the effective Hamiltonian H_{int} mentioned in the main context, its

eigenvalues and eigenvectors are obtained as $\lambda = \pm(\delta^2 - \chi^2)^{1/2}$, and $[\psi_1^{\mathrm{nf}}, \psi_2^{\mathrm{nf}}]^{\mathrm{T}} = [1, -i(\delta \pm (\delta^2 - \chi^2)^{1/2})/\chi)]^{\mathrm{T}}$. The eigenvectors are discussed in the following:

1. $\delta > \chi$

$$|\frac{\psi_2^{\mathrm{nf}}}{\psi_1^{\mathrm{nf}}}| = \frac{\delta \pm \sqrt{\delta^2 - \chi^2}}{|\chi|}$$

$$\arctan\left(\frac{\psi_2^{\mathrm{nf}}}{\psi_1^{\mathrm{nf}}}\right) = -\frac{\pi}{2}\ (\chi > 0) \text{ or } \frac{\pi}{2}\ (\chi < 0)$$

2. $\delta < \chi$

$$|\frac{\psi_2^{\mathrm{nf}}}{\psi_1^{\mathrm{nf}}}| = 1$$

$$\arctan\left(\frac{\psi_2^{\mathrm{nf}}}{\psi_1^{\mathrm{nf}}}\right) = \frac{\delta}{\mp\sqrt{\chi^2 - \delta^2}}$$

The condition $\delta < \chi$ gives the APT-S phase, whose corresponding eigenstates exhibit the unit amplitude ratio and varied phase differences. While $\delta > \chi$ indicates the APT-B phase, whose corresponding eigenstates exhibit non-unit amplitude ratios with a fixed phase difference.

Supplementary Note IV

Parameter Extraction

1. Frequency detunings $\omega_0\delta$

Regarding a single DSPR, the excitation-probe setup (shown in Fig. S3a) can be described as equations in the following:

$$\begin{cases} \omega\psi = (\omega_0 + i\gamma_0)\psi + \eta s \\ p = \eta\psi \end{cases} \tag{4.1}$$

where ω_0 and γ_0 represent the eigenfrequency and decay rate of the resonant mode ψ respectively. η indicates the coupling efficiency between the resonant mode and the source (s) or probe (p). Solving the equations, the transmission coefficient is obtained as

$$t = \left|\frac{p}{s}\right| = |\frac{\eta^2}{\omega - \omega_0 - i\gamma_0}| \tag{4.2}$$

which satisfies the Lorentzian form. Therefore, we change the inner radius r of the DSPR as 6, 6.3, 6.7, 7.0, 7.3 mm respectively, and the transmission spectra are shown

in Fig. S3a. By Lorentz fitting, their corresponding eigenfrequencies ω_1 are extracted as 3.625, 3.675, 3.747, 3.795, 3.848 GHz. ω_2 is 3.625 GHz since we keep the inner radius r = 6 mm for the left DSPR. Consequently, the frequency detuning parameters $\omega_0\delta$ of the above five samples are extracted as 0, 0.025, 0.061, 0.085, and 0.1115 GHz.

2. Coupling strength $\omega_0\chi$

Eq.4.1 can be extended to a dimer system (shown in the inset of Fig. S3b) as following

$$\begin{cases} \omega \begin{bmatrix} \psi_1^{\mathrm{nf}} \\ \psi_2^{\mathrm{nf}} \end{bmatrix} = \begin{bmatrix} \omega_0 + i\gamma_0 - \omega_0\delta & i\omega_0\chi \\ i\omega_0\chi & \omega_0 + i\gamma_0 + \omega_0\delta \end{bmatrix} \begin{bmatrix} \psi_1^{\mathrm{nf}} \\ \psi_2^{\mathrm{nf}} \end{bmatrix} + \begin{bmatrix} \eta s \\ 0 \end{bmatrix} \\ p = \eta\psi_2^{\mathrm{nf}} \end{cases} \tag{4.3}$$

By simple manipulation, we can also obtain the near-field transmission coefficient

$$t = \left|\frac{p}{s}\right| = \left| \frac{i\chi\eta^2}{2\sqrt{\delta^2-\chi^2}} \left(\frac{1}{\omega-\omega_0-i\gamma_0-\omega_0\sqrt{\delta^2-\chi^2}} - \frac{1}{\omega-\omega_0-i\gamma_0+\omega_0\sqrt{\delta^2-\chi^2}} \right) \right| \tag{4.4}$$

Therefore, by fitting the simulated/measured transmission spectra with Eq.4.4, we can extract the indirect coupling factor $\omega_0\chi$ of odd modes as 0.025 GHz.

Supplementary Note V

Experimental setup

The near-field and far-field experimental setups are shown in Fig. S4a-b. Experimental measurements are carried out in the microwave anechoic chamber using the vector network analyzer (VNA). In the near-field experiments, the sample is excited by the near-field source, and the probe is vertically suspended on a 2D moving stage controlled by the computer to capture the field patterns by detecting E_z components, avoiding interference from the incident waves. In the far-field experiments, we use the horn antenna as the far-field excitation source, and the probe is also vertically suspended on the 2D moving stage. It is worth noting that the odd mode of DSPRs is excited when the electric field of incident waves is along the x direction, while plane waves with the electric field along the y direction excite the even mode.

Supplementary Note VI

The excited modes with near/far-field excitation

To elaborate on the deviations between the excited modes and eigenmodes, we utilize a generalized coupled-mode equation with driving terms as follows:

$$\omega \begin{bmatrix} \psi_1^{\mathrm{nf}} \\ \psi_2^{\mathrm{nf}} \end{bmatrix} = \begin{bmatrix} \omega_0 + i\gamma_0 - \omega_0\delta & i\omega_0\chi \\ i\omega_0\chi & \omega_0 + i\gamma_0 + \omega_0\delta \end{bmatrix} \begin{bmatrix} \psi_1^{\mathrm{nf}} \\ \psi_2^{\mathrm{nf}} \end{bmatrix} + \begin{bmatrix} \alpha s \\ \beta s \end{bmatrix} \tag{6.1}$$

where α (β) indicates the coupling efficiency between the resonant mode $\psi_{1(2)}{}^{\mathrm{nf}}$ and the source s. Solving the coupled-mode equations, the excited modes are obtained as

$$\begin{bmatrix} \psi_1^{\mathrm{nf}} \\ \psi_2^{\mathrm{nf}} \end{bmatrix} = \frac{i}{[\omega-(\omega_0-\omega_0\delta+i\gamma_0)][\omega-(\omega_0+\omega_0\delta+i\gamma_0)]+(\omega_0\chi)^2} \begin{bmatrix} [\omega-(\omega_0+\omega_0\delta+i\gamma_0)]\alpha s + i\omega_0\chi\beta s \\ [\omega-(\omega_0-\omega_0\delta+i\gamma_0)]\beta s + i\omega_0\chi\alpha s \end{bmatrix} \tag{6.2}$$

Therefore, the amplitude ratio is derived as

$$\frac{\psi_2^{\mathrm{nf}}}{\psi_1^{\mathrm{nf}}} = \frac{(\Delta - i\gamma_0 + \omega_0\delta)\beta + i\omega_0\chi\alpha}{(\Delta - i\gamma_0 - \omega_0\delta)\alpha + i\omega_0\chi\beta} \tag{6.3}$$

where $\Delta = \omega - \omega_0$.

When the system is excited by a near-field point source, we describe the sources as $[\alpha, \beta] = [1, 0]$ (left) and $[\alpha, \beta] = [0, 1]$ (right), which are determined by the position of the source. While under normal illuminations of plane waves, we describe the source as $[\alpha, \beta] = [1, 1]$ due to the symmetrical excitation of the two resonators.

At the exceptional point $\delta = \chi$ and resonance frequency $\omega = \omega_0$, the amplitude ratio is discussed as follows:

1. $[\alpha, \beta] = [1, 0]$

$$\frac{\psi_2^{\mathrm{nf}}}{\psi_1^{\mathrm{nf}}} = \frac{i\omega_0\chi}{\Delta - i\gamma_0 - \omega_0\delta} = \frac{i\omega_0\delta}{-i\gamma_0 - \omega_0\delta}$$

$$\left|\frac{\psi_2^{\mathrm{nf}}}{\psi_1^{\mathrm{nf}}}\right| = \sqrt{\frac{(\omega_0\delta)^2}{\gamma_0{}^2 + (\omega_0\delta)^2}}$$

2. $[\alpha, \beta] = [0, 1]$

$$\frac{\psi_2^{\mathrm{nf}}}{\psi_1^{\mathrm{nf}}} = \frac{\Delta - i\gamma_0 + \omega_0\delta}{i\omega_0\chi} = \frac{-i\gamma_0 + \omega_0\delta}{i\omega_0\delta}$$

$$\left|\frac{\psi_2^{\mathrm{nf}}}{\psi_1^{\mathrm{nf}}}\right| = \sqrt{\frac{\gamma_0{}^2 + (\omega_0\delta)^2}{(\omega_0\delta)^2}}$$

3. $[\alpha, \beta] = [1, 1]$

$$\frac{\psi_2^{\mathrm{nf}}}{\psi_1^{\mathrm{nf}}} = \frac{-i\gamma_0 + \omega_0\delta + i\omega_0\delta}{-i\gamma_0 - \omega_0\delta + i\omega_0\delta}$$

$$\left|\frac{\psi_2^{\mathrm{nf}}}{\psi_1^{\mathrm{nf}}}\right| = 1$$

Therefore, looking into the near-field excited mode at EP, i.e. $[\psi_1^{\mathrm{nf}}, \psi_2^{\mathrm{nf}}]^{\mathrm{T}} = (\gamma_0/2 - i\omega_0\delta)[1, -i]^{\mathrm{T}} + \gamma_0/2[1, i]^{\mathrm{T}}$, the deviation from the calculated eigenmode $[1, -i]^{\mathrm{T}}$ is due to the emergence of the missing eigenstate by asymmetric excitation in the presence of the background loss γ_0. For similar reasons, the phase difference of the far-field excited mode deviates slightly from π/2, which is obtained as $[\psi_1^{\mathrm{nf}}, \psi_2^{\mathrm{nf}}]^{\mathrm{T}} = (-i\gamma_0/2 - \gamma_0/2)[1, i]^{\mathrm{T}} + (-i\gamma_0/2 + \gamma_0/2 + i\omega_0\delta - \omega_0\delta)[1, -i]^{\mathrm{T}}$.

In the APT-S phase ($\delta = 0$), the amplitude ratio is reduced as following

1. $[\alpha, \beta] = [1, 0]$

$$\frac{\psi_2^{\mathrm{nf}}}{\psi_1^{\mathrm{nf}}} = \frac{\omega_0\chi}{-\gamma_0}$$

$$\left|\frac{\psi_2^{\mathrm{nf}}}{\psi_1^{\mathrm{nf}}}\right| = \left|\frac{\omega_0\chi}{\gamma_0}\right|$$

2. $[\alpha, \beta] = [0, 1]$

$$\frac{\psi_2^{\mathrm{nf}}}{\psi_1^{\mathrm{nf}}} = \frac{-\gamma_0}{\omega_0\chi}$$

$$\left|\frac{\psi_2^{\mathrm{nf}}}{\psi_1^{\mathrm{nf}}}\right| = \left|\frac{\gamma_0}{\omega_0\chi}\right|$$

3. $[\alpha, \beta] = [1, 1]$

$$\frac{\psi_2^{\mathrm{nf}}}{\psi_1^{\mathrm{nf}}} = 1$$

Therefore, the calculated excited mode in the APT-S phase with near-field excitation is obtained as a superposition of the eigenmodes with different weights, i.e. $[\psi_1^{\mathrm{nf}}, \psi_2^{\mathrm{nf}}]^{\mathrm{T}} = (\omega_0\chi/2 + \gamma_0/2)[1, -1]^{\mathrm{T}} + (\omega_0\chi/2 - \gamma_0/2)[1, 1]^{\mathrm{T}}$. The high-Q mode $[1, -1]^{\mathrm{T}}$ is excited with a larger efficiency $(\omega_0\chi + \gamma_0)/2$, thus dominating the measured near-field spectrum and pattern. Moreover, the far-field excited mode in the APT-S phase shows a pure $[1, 1]^{\mathrm{T}}$ mode, which is consistent with the field pattern in Fig. 4d.

In the APT-B phase, the amplitude ratio is reduced as follows at the resonance frequency $\omega = \omega_0 \pm \omega_0(\delta^2 - \chi^2)^{1/2}$:

1. $[\alpha, \beta] = [1, 0]$

$$\frac{\psi_2^{\mathrm{nf}}}{\psi_1^{\mathrm{nf}}} = \frac{i\omega_0\chi}{\left(\pm\,\omega_0\sqrt{\delta^2-\chi^2} - i\gamma_0 - \omega_0\delta\right)}$$

$$\left|\frac{\psi_2^{\mathrm{nf}}}{\psi_1^{\mathrm{nf}}}\right| = \sqrt{\frac{(\omega_0\chi)^2}{{\gamma_0}^2 + \omega_0(\pm\sqrt{\delta^2-\chi^2} - \delta)^2}}$$

2. $[\alpha, \beta] = [0, 1]$

$$\frac{\psi_2^{\mathrm{nf}}}{\psi_1^{\mathrm{nf}}} = \frac{\left(\pm\,\omega_0\sqrt{\delta^2-\chi^2} - i\gamma_0 + \omega_0\delta\right)}{i\omega_0\chi}$$

$$\left|\frac{\psi_2^{\mathrm{nf}}}{\psi_1^{\mathrm{nf}}}\right| = \sqrt{\frac{{\gamma_0}^2 + \omega_0(\pm\sqrt{\delta^2-\chi^2} + \delta)^2}{(\omega_0\chi)^2}}$$

3. $[\alpha, \beta] = [1, 1]$

$$\frac{\psi_2^{\mathrm{nf}}}{\psi_1^{\mathrm{nf}}} = \frac{\left(\pm\,\omega_0\sqrt{\delta^2-\chi^2} - i\gamma_0 + \omega_0\delta\right) + i\omega_0\chi}{\left(\pm\,\omega_0\sqrt{\delta^2-\chi^2} - i\gamma_0 - \omega_0\delta\right) + i\omega_0\chi}$$

$$\left|\frac{\psi_2^{\mathrm{nf}}}{\psi_1^{\mathrm{nf}}}\right| = \sqrt{\frac{(\omega_0\chi - \gamma_0)^2 + {\omega_0}^2(\pm\sqrt{\delta^2-\chi^2} + \delta)^2}{(\omega_0\chi - \gamma_0)^2 + {\omega_0}^2(\pm\sqrt{\delta^2-\chi^2} - \delta)^2}}$$

Under normal illuminations of plane waves, the lower-frequency excitation gives stronger fields on the down-detuned resonator, while higher-frequency excitation gives stronger fields on the up-detuned one. Therefore, such properties in the APT-B phase are promising in frequency sensing.

Supplementary Note VII

The detuning-induced APT phase transition under space-wave illuminations

Besides near-field demonstration of the APT phase transition by adjusting the detuning, the detuning-induced APT phase transition is further observed under space-wave illuminations attributed to the radiative property. The same samples with different radii (r_R = 6, 6.3, 6.7, 7.0, 7.3 mm) are used in experiments as in the near-field demonstration (the far-field experimental setup is shown in SMV).

The experimental resonance frequencies are marked as red stars in Fig. S5a, which are consistent with both calculated results and near-field excited spectra. The far-field excited field patterns on the samples of $|\delta/\chi| = 0$ (APT-S) and 4.46 (APT-B) exhibit unit and non-unit amplitude ratios respectively, and further verify the APT phase transition. Interestingly, the captured field pattern on the APT-S sample ($|\delta/\chi| = 0$) shows a pure low-Q mode $[1, 1]^T$ (Fig. S5b), without any sign of the high-Q mode $[1, -1]^T$. Since the normally incident space wave is described as a symmetric source $[\alpha, \beta] = [1, 1]$, it excites the symmetric mode $[1, 1]^T$ selectively (see Supplementary Information VI for details), but cannot excite the orthogonal mode $[1, -1]^T$. The non-unit field patterns (Fig. S5d-e) on the APT-B sample are consistent with both calculated eigenstates and near-field excited patterns. Moreover, the excited mode in the APT-B phase is obtained as:

$|\psi_2^{nf}/\psi_1^{nf}| = \sqrt{[(\omega_0\chi-\gamma_0)^2+(\Delta+\omega_0\delta)^2]/[(\omega_0\chi-\gamma_0)^2+(\Delta-\omega_0\delta)^2]}$ where $\Delta = \omega - \omega_0$. The lower-frequency ($\omega < \omega_0$) excitation gives stronger fields on the down-detuned resonator, while higher-frequency ($\omega > \omega_0$) gives stronger fields on the up-detuned one. Such properties in the APT-B phase are promising in frequency sensing. Regarding the EP sample of $|\delta/\chi| = 1$, the captured field pattern (shown in Fig. S5c) corresponds well to the calculated eigenstate, except that the phase difference deviates slightly from π/2. Such deviation is also attributed to the emergence of the missing eigenstate excited by the far-field source in the presence of the background loss γ_0 (see Supplementary Information VI for details).

Supplementary Note VIII

Detection of environmental variation based on APT systems

Here the designed APT system is utilized for the near-field detection of environmental variations, i.e. the permittivity of the environmental media.

We consider the case that the left DSPR is covered by the media with a relative permittivity ε_r and keep the other parts of the system unchanged (shown in Fig. S6a). When the permittivity of the environmental media changes, the resonant frequency of

the left DSPR changes, further leading to the change of the frequency detuning. It means that the APT-phase transition can be realized by changing the permittivity of the environmental media.

Based on the above analysis, we simulate the near-field transmission spectra as shown in Fig. S6b when ε_r = 1, 1.5, 2 and 3. From the figure, the resonant peak of the transmission spectra splits with the increase of ε_r, which means the system evolves from the APT-S phase to APT-B phase. Therefore, we can detect the environmental variations according to the resonance-frequency split of the APT system.

Supplementary Note IX

Remote sensing of environmental variations

The radiative feature of our system also enables us to observe the APT phase transition in the far field by using the environmental-index-tuning approach. In other words, we can remotely sense the changing of the environmental index, using the radiative APT systems.

We use the same sample and tuning approach here as in the near-field sensing (see Supplementary Information VIII for details). The sample is illuminated with H-polarized space waves, and we use reflection spectra to observe the manifestation of the radiative APT system (shown in Fig. S7a).

The simulated reflection spectra are shown in Fig. S7b when ε_r = 1, 2 and 3. From the figure, the resonant peak of the transmission spectra splits with the increase of ε_r, which means the system evolves from the APT-S phase to the APT-B phase. Therefore, we can remotely sense the environmental variations according to the reflection spectra of our radiative APT system. These results confirm that our radiative system is promising in constructing APT-empowered radiative devices and remote sensing applications.

Supplementary Note X

Eigen solutions of the higher-order APT Hamiltonian

The higher-order APT Hamiltonian is expressed as $H = \omega_0 + i\gamma_0 + \omega_0 H_4$.

$$H_4 = \begin{bmatrix} -\delta & i\chi & 0 & 0 \\ i\chi & \delta & i\chi & 0 \\ 0 & i\chi & -\delta & i\chi \\ 0 & 0 & i\chi & \delta \end{bmatrix}$$

Solving the higher-order APT Hamiltonian H_4, its eigenvalues $\lambda_{1,2,3,4}$ and corresponding eigenvectors $[\psi_1^{nf}, \psi_2^{nf}, \psi_3^{nf}, \psi_4^{nf}]^T_{1,2,3,4}$ are obtained as below:

$$\lambda_{1,2,3,4} = \{-\sqrt{\frac{2\delta^2+(-\sqrt{5}-3)\chi^2}{2}}, \sqrt{\frac{2\delta^2+(-\sqrt{5}-3)\chi^2}{2}}, -\sqrt{\frac{2\delta^2+(\sqrt{5}-3)\chi^2}{2}}, \sqrt{\frac{2\delta^2+(\sqrt{5}-3)\chi^2}{2}}\}$$

$$[\psi_1^{nf}, \psi_2^{nf}, \psi_3^{nf}, \psi_4^{nf}]_1^T = \left[\frac{i(\sqrt{5}-1)\left(2\delta+\sqrt{2}\sqrt{2\delta^2+(-\sqrt{5}-3)\chi^2}\right)}{4\chi}, \frac{1+\sqrt{5}}{2}, \frac{i\left(2\delta+\sqrt{2}\sqrt{2\delta^2+(-\sqrt{5}-3)\chi^2}\right)}{2\chi}, 1\right]^T$$

$$[\psi_1^{nf}, \psi_2^{nf}, \psi_3^{nf}, \psi_4^{nf}]_2^T = \left[-\frac{i(\sqrt{5}-1)\left(-2\delta+\sqrt{2}\sqrt{2\delta^2-(\sqrt{5}+3)\chi^2}\right)}{4\chi}, \frac{1+\sqrt{5}}{2}, -\frac{i\left(-2\delta+\sqrt{2}\sqrt{2\delta^2-(\sqrt{5}+3)\chi^2}\right)}{2\chi}, 1\right]$$

$$[\psi_1^{nf}, \psi_2^{nf}, \psi_3^{nf}, \psi_4^{nf}]_3^T = \left[-\frac{i(\sqrt{5}+1)\left(2\delta+\sqrt{2}\sqrt{2\delta^2+(\sqrt{5}-3)\chi^2}\right)}{4\chi}, \frac{1-\sqrt{5}}{2}, \frac{i\left(2\delta+\sqrt{2}\sqrt{2\delta^2+(\sqrt{5}-3)\chi^2}\right)}{2\chi}, 1\right]$$

$$[\psi_1^{nf}, \psi_2^{nf}, \psi_3^{nf}, \psi_4^{nf}]_4^T = \left[\frac{i(\sqrt{5}+1)\left(-2\delta+\sqrt{2}\sqrt{2\delta^2+(\sqrt{5}-3)\chi^2}\right)}{4\chi}, \frac{1-\sqrt{5}}{2}, -\frac{i\left(-2\delta+\sqrt{2}\sqrt{2\delta^2+(\sqrt{5}-3)\chi^2}\right)}{2\chi}, 1\right]$$

According to the theoretical results, the higher-order APT system undergoes the two-stage APT phase transition during sweeping the frequency detuning δ. The eigenvectors in each APT phase are discussed in detail as follows:

1. APT-S: $0 \leq \delta \leq (\sqrt{5}-1)\chi/2$

$$[|\psi_1^{nf}|, |\psi_2^{nf}|, |\psi_3^{nf}|, |\psi_4^{nf}|]_1 = [|\psi_1^{nf}|, |\psi_2^{nf}|, |\psi_3^{nf}|, |\psi_4^{nf}|]_2 = \left[1, \frac{1+\sqrt{5}}{2}, \frac{1+\sqrt{5}}{2}, 1\right]$$

$$[|\psi_1^{nf}|, |\psi_2^{nf}|, |\psi_3^{nf}|, |\psi_4^{nf}|]_3 = [|\psi_1^{nf}|, |\psi_2^{nf}|, |\psi_3^{nf}|, |\psi_4^{nf}|]_4 = \left[1, \frac{\sqrt{5}-1}{2}, \frac{\sqrt{5}-1}{2}, 1\right]$$

2. 1st-APT-B: $(\sqrt{5}-1)\chi/2 \leq \delta \leq (1+\sqrt{5})\chi/2$

$$[|\psi_1^{nf}|, |\psi_2^{nf}|, |\psi_3^{nf}|, |\psi_4^{nf}|]_1 = [|\psi_1^{nf}|, |\psi_2^{nf}|, |\psi_3^{nf}|, |\psi_4^{nf}|]_2 = \left[1, \frac{1+\sqrt{5}}{2}, \frac{1+\sqrt{5}}{2}, 1\right]$$

$$\text{phase}(\psi_1^{nf}, \psi_2^{nf}, \psi_3^{nf}, \psi_4^{nf})_3 = \text{phase}(\psi_1^{nf}, \psi_2^{nf}, \psi_3^{nf}, \psi_4^{nf})_4 = \left[\frac{3\pi}{2}, \pi, \frac{\pi}{2}, 0\right] (\chi > 0) \text{ or } \left[-\frac{3\pi}{2}, -\pi, -\frac{\pi}{2}, 0\right] (\chi < 0)$$

3. 2nd-APT-B: $\delta \geq (1+\sqrt{5})\chi/2$

$$\text{phase}(\psi_1^{nf}, \psi_2^{nf}, \psi_3^{nf}, \psi_4^{nf})_1 = \text{phase}(\psi_1^{nf}, \psi_2^{nf}, \psi_3^{nf}, \psi_4^{nf})_2 = \left[\frac{\pi}{2}, 0, \frac{\pi}{2}, 0\right] (\chi > 0) \text{ or } \left[-\frac{\pi}{2}, 0, -\frac{\pi}{2}, 0\right] (\chi < 0)$$

$$\text{phase}(\psi_1^{nf}, \psi_2^{nf}, \psi_3^{nf}, \psi_4^{nf})_3 = \text{phase}(\psi_1^{nf}, \psi_2^{nf}, \psi_3^{nf}, \psi_4^{nf})_4 = \left[\frac{3\pi}{2}, \pi, \frac{\pi}{2}, 0\right] (\chi > 0) \text{ or } \left[-\frac{3\pi}{2}, -\pi, -\frac{\pi}{2}, 0\right] (\chi < 0)$$

Firstly, the condition $|\delta/\chi| < (\sqrt{5}-1)/2$ gives the high-order APT-S phase, whose corresponding eigenvalues λ are four pure imaginary values and the eigenstates exhibit the fixed amplitude ratios, i.e. $|\psi_1^{nf}| : |\psi_2^{nf}| : |\psi_3^{nf}| : |\psi_4^{nf}| = 1 : (\sqrt{5}+1)/2 : (\sqrt{5}+1)/2 : 1$ as Group+ and $1 : (\sqrt{5}-1)/2 : (\sqrt{5}-1)/2 : 1$ as Group-. Secondly, the system is in the 1st-APT-B phase when $(\sqrt{5}-1)/2 < |\delta/\chi| < (\sqrt{5}+1)/2$. The eigenvalues are two real values with eigenvectors in Group- of the fixed phase difference $[\pm3\pi/2, \pm\pi, \pm\pi/2, 0]$, and two imaginary values with eigenvectors in Group+ of the fixed amplitude ratio $[1, (\sqrt{5}+1)/2, (\sqrt{5}+1)/2, 1]$. While $|\delta/\chi| > (\sqrt{5}+1)/2$ indicates the 2nd-APT-B phase, whose corresponding eigenvalues are all pure real values, and eigenstates exhibit varying amplitude ratios with the fixed phase differences ($[\pm3\pi/2, \pm\pi, \pm\pi/2, 0]$ in Group- and $[\pm\pi/2, \pm\pi, \pm\pi/2, 0]$ in Group+). $|\delta/\chi| = (\sqrt{5}-1)/2$ and $|\delta/\chi| = (\sqrt{5}+1)/2$ are EPs marking the two-stage APT phase transition.

Supplementary Note XI

The extension of APT design to the optical frequency

Here we show the extension capability of our plasmonic APT design. Our design mechanism of APT systems is not limited to microwave frequencies. It can be further extended to optical frequencies, and the extension approach lies in the following two aspects:

1. The leaky-wave mechanism extension

The system (dielectric on metal) can host surface EM waves in both microwave and optical frequencies, and also host corresponding leaky waves above the light line. The only difference is the metal properties in the two frequency regions. In microwave, metal approximately behaves as a perfect electric conductor (PEC), while in optics it follows the dispersion of the Drude model. Despite the existence of such a difference, leaky waves exist in both frequency regions.

2. The plasmonic resonator extension

In optical frequency, plasmonic-resonator extension is also feasible. Since the DSPRs are low-frequency analogous to the metallic nanodisks at optical frequencies[3].

To achieve the extension, we replace the textured metal disk in the microwave with metallic nanodisks in optics.

Conclusively, with the same configuration and mechanism, our designed architecture can be extended to optical frequencies. As shown in Fig. S8a, the APT system consists of two silver (Ag) nanodisks with the dielectric layer and metal substrate (Ag) at the bottom. The nanodisk also supports a tightly confined dipole mode and the horizontal decay length is L_x = 70 nm from the edge of the disk. The background structure hosts horizontally propagating waves, which radiate into free space by the out-of-plane leakage, providing the indirect coupling channels. We set the edge-edge distance of the two disks sufficiently distant as d = 170 nm, which is larger than $2L_x$.

To verify the symmetry-breaking process, we design samples with different frequency detunings. We change the inner radius r_{L} of the left DSPR as 60, 57.5, and 56.5 nm respectively, while keeping the r_{R} = 60 nm for the right DSPR.

The simulated spectra are shown in Fig. S8b. When r_{L} = 60 nm, the frequency detuning parameter $\omega_0\delta$ is extracted as 0, which corresponds to the APT-S phase. Thus, the simulated transmission spectrum only has one peak. The low-Q eigenmode [1, 1] and the high-Q eigenmode [1, -1] are excited by the far-field (Fig. S8c) and near-field (Fig. S8d) source, respectively. Due to the effect of the near-field source, the amplitude of the high-Q eigenmode slightly deviates from the calculated result. When r_{L} = 56.5 nm, the simulated transmission spectrum splits, which corresponds to the APT-B phase. The low-frequency eigenmode (Fig. S8e) and high-frequency eigenmode (Fig. S8f) have the same phase difference -π/2, which is consistent with the theoretical analysis.

Supplementary Note XII

Effect of the substrate thickness on imaginary couplings

In our APT system, the thickness h of the dielectric substrate is elaborately designed to ensure efficient imaginary coupling by leaky waves.

The strength of imaginary coupling decreases as h decreases, according to the theoretical expression $i\chi \propto h\cdot\int(\psi_{1(2)}^{\mathrm{nf}}\cdot \psi_{2(1)}^{\mathrm{p}})/(|C_{\mathrm{NF}}|\cdot|C_{\mathrm{FF}}|)\mathrm{d}x\mathrm{d}y$ (in the main text). This relation is also verified with simulations (Fig. S9). When the substrate is too thin, the leaky wave coupling will tend to vanish. In this scenario, the new system is composed of two independent high-Q resonators, and does not show any APT features. Also, the new system hardly interacts with space waves.

Supplementary Note XIII

The unique physical properties of the leaky-wave coupling

To show the unique physical properties of the leaky-wave coupling, we compare the leaky wave with the atomic coherence leakage[1] in Table S1 and Fig. S10a. The leakage of leaky waves refers to the EM wave leaking from the in-plane guided modes. While the atomic coherence leakage refers to the flying atoms escaping from the atomic spin waves.

We further elucidate the uniqueness of leaky waves with its concept. It is a type of special radiative EM mode derived from guided modes without radiation leakage. Fig. S10 b-c show the typical structure and field pattern of leaky waves. The typical structure consists of a dielectric layer coated with the conductor (in Fig. S10b). Such the structure can host guided modes without radiation leakage, which propagate along the z direction and decay in the air along the x direction. Thus, its wavenumber is obtained as $\mathbf{k}_{\mathbf{NR}} = \mathbf{z}\beta_{\mathrm{nz}} + \mathbf{x}i\alpha_{\mathrm{nz}}$, where β_{nz} and α_{nz} denote the propagation constant and attenuation rate, respectively. Small perturbations can derive these non-radiative guided modes into radiative leaky modes, which radiate into the air along the x direction. The wavenumber of the radiative leaky mode is $\mathbf{k}_{\mathbf{R}} = \mathbf{z}(\beta_{\mathrm{z}} + i\alpha_{\mathrm{z}}) + \mathbf{x}(\beta_{\mathrm{x}} - i\alpha_{\mathrm{x}})$, which satisfies the dispersion relation $(\omega/c)^2 = (\beta_{\mathrm{z}} + i\alpha_{\mathrm{z}})^2 + (\beta_{\mathrm{x}} - i\alpha_{\mathrm{x}})^2$. The corresponding field pattern is shown in Fig. S10c.

Reference

1. Peng, P., et al. Anti-parity-time symmetry with flying atoms. *Nat. Phys*. **12**, 1139 (2016).
2. Wonjoo, S., Zheng, W., &Shanhui, F. Temporal coupled-mode theory and the presence of non-orthogonal modes in lossless multimode cavities. *IEEE J Quantum Electron* **40**, 1511-1518 (2004).
3. Garcia-Vidal, F. J., et al. Spoof surface plasmon photonics. *Rev. Mod. Phys*. **94**, 025004 (2022).
